# Pedunculate leiomyoma of the scrotum

**DOI:** 10.11604/pamj.2015.20.447.6817

**Published:** 2015-04-30

**Authors:** Benhiba Hind, Hassam Badredine

**Affiliations:** 1 Department of Dermatology-venerology, Ibn Sina Hospital, University Mohammed V, Souissi, Rabat, Morocco

**Keywords:** Leiomyoma, scrotum, tumor

## Image in medicine

Leiomyoma originating from the scrotum is a scarce entity. We report a case of scrotal leiomyoma which was unique to us. A 35 year-old man sought medical care for a painless scrotal lesion that had enlarged over a 3-year period and was causing him discomfort. His medical history was otherwise unremarkable: there was no history of trauma, inflammation, or infection, and no significant urologic antecedent. Physical examination revealed a 2x2 cm painless skin-colored nodule of rubbery consistency on the left side of the scrotum, which was well circumscribed, pedunculate and clearly separate from the testicles and epididymis. There was no inguinal lymphadenopathy. Scrotal ultrasonography demonstrated that the scrotal contents were normal. Under local anesthesia, the scrotal mass and surrounding skin were surgically resected. Histologic examination was consistent with leiomyoma. There was no evidence of cyto-nuclear atypia. The patient's postoperative recovery was uneventful. On follow-up visit 10 days later, he did not have any complications, and the sutures were removed. At his last visit 12 months after the procedure, he was still asymptomatic and did not have any recurrence. Leiomyoma is a benign tumor of smooth muscles that may arise anywhere in the body. Smooth muscle tumors of scrotum were first described by Forsters in 1858. They arise from the dartos muscle and are extremely rare: less than 50 cases were reported in the literatures. Patients usually present with painless solitary small cutaneous lesion, and that's histology which confirms the diagnosis. Treatment is based on a simple surgical excision. Exceedingly rare, scrotal leiomyoma are usually misdiagnosed. Thus, this entity should be suspected in cases of scrotal masses.

**Figure 1:**
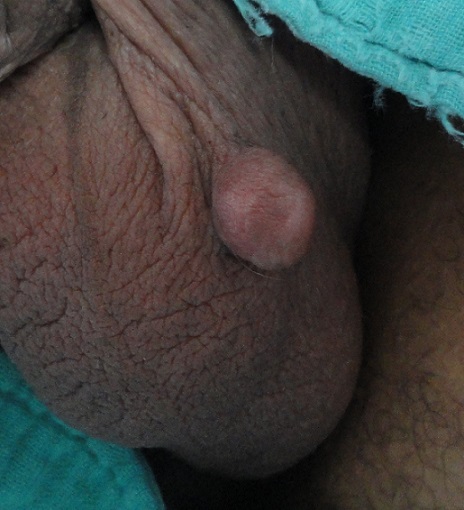
Pedunculate nodule of the scrotum

